# Forms of Albumen in the Urine, and Their Tests

**Published:** 1887-08

**Authors:** 


### THE FORMS OF ALBUMEN IN THE URINE, AND THEIR TESTS.

DR. T. GRAINGER STEWART, physician in Ordinary to the Queen for Scotland, in a lecture at the University of Edinburgh, gave the following account of the forms of albumen found in the urine, and the results of a careful and prolonged inquiry which, along with Dr. Stevens, he has made as to the comparative delicacy of the leading tests for serum albumen, and as to the value of the different methods of quantitative analysis by which the amount of albumen may be determined.

The fôrms of albumen met with in the urine are :—

I. *Serum Albumen,* a substance which, according to Hammarsten, constitutes 4.516 per cent. of the blood serum. It is almost constantly present in urine which contains any variety of albumen. Although a less diffusible body than serum globulin, it is capable of passing through membrane.

II. *Serum Globulin or Paraglobulin,* the globulin of the blood serum, of which it constitutes 3.103 per cent. It is met with in almost all albuminous urines, its proportion to the serum albumen varying in different instances.

III. *Peptone,* a product of gastric and pancreatic digestion of albuminous substances, also occuring in the process of transformation of tissues and of inflammatory effusions. It is a readily diffusible substance, occasionally met with in the urine in association with or apart from serum albumen.

IV. *Propeptone, or Parapeptone, or Hemialbumose,* a substance or group of substances intermediate between albumen and peptone, constituting a stage or stages of transformation from the one to the other. It is highly diffusible, and is occasionally met with in the urine under conditions corresponding to those under which peptone occurs. This is the peculiar form of albumen which was discovered in the urine by Dr. Bence Jones in a case of osteomalacia.

V. *Acid Albumen, or Syntonin,* one of the derived proteids obtained by the action of acids upon albumen. It is easily produced artificially by the addition of acid to albuminous urine, but may occur naturally in certain cases.

VI. *Alkali Albumen,* another derived proteid, produced by the action of alkalies upon albumen. It is readily produced artificially, but is also found naturally in the urine.

VII. *Hæmoglobin*, the combination of hæmatin and globulin naturally existing in the red corpuscles of the blood. It sometimes appears in the urine, particularly in cases of hæmaturia and hæmoglobinuria, also in certain septic conditions, and after inhalation of arseniuretted hydrogen, transfusion of blood, and otherwise.

VIII. *Fibrin*, a proteid substance which does not normally exist as such in the blood. It is met with in the urine in hæmaturia, in some cases of chyluria, and in certain varieties of renal casts.

IX. *Mucin*, the chief constituent of mucus, is a derived proteid substance. It frequently becomes superadded to the urine after secretion, and may be derived from any part of the urinary tract.

X. *Lardacein, Waxy or Amyloid Material*, familiarly known as a pathological substance within the body, is said to be occasionally demonstrable in renal casts.

Of these ten varieties the last four are of little practical importance—mucin alone being indeed worthy of special comment, and that mainly because of the difficulties which its presence raises in regard to the reliability of certain tests for serum albumen.

As to the tests for the albumens, he puts in tabular form the chief tests for the different varieties of albumen, with their actions upon each variety.

TABLE I.—SHOWING TESTS FOR THE CHIEF FORMS OF ALBUMEN.

| | *Serum Albumen.* | *Serum Globulin.* | *Peptones.* | *Propeptones.* | *Acid Albumen.* | *Alkali Albumen.* |
|---|---|---|---|---|---|---|
| Heat. | | | | | | 0 |
| Heat with nitric acid. / Heat with acetic acid. | Opacity. | Opacity. | 0 | 0 | 0 | Opacity. |
| Cold, nitric acid. | Opacity. | Opacity. | 0 | Opacity dissolved by heat. | Opacity. | Opacity. |
| Metaphosphoric acid. | Opacity. | Opacity. | Opacity diminished or dissolved by heat. | Opacity diminished or dissolved by heat. | 0 | Opacity. |
| Acidulated brine. | Opacity. | Opacity. | Opacity diminished or dissolved by heat. | Opacity diminished or dissolved by heat. | Opacity. | Opacity. |
| Picric acid. | Opacity. | Opacity. | Opacity dissolved by heat. | Opacity dissolved by heat. | Opacity. | Opacity. |
| Potassio-mercuric-iodide. | Opacity. | Opacity. | Opacity dissolved by heat. | Opacity dissolved by heat. | Opacity. | Opacity. |
| Potassium ferrocyanide. | Opacity. | Opacity. | 0 | Opacity dissolved by heat. | Opacity. | Opacity. |
| Dilution with water. | 0 | Slight opacity. | 0 | 0 | 0 | 0 |
| Magnesium sulphate. | 0 | Opacity. | 0 | 0 | Opacity. | Opacity. |
| Fehling's solution. | Brownish-red or mauve. | -- | Rose pink or purple. | Rose pink or purple. | | |
| Randolph's test. | -- | -- | Yellow opacity. | Yellow opacity. | | |

The oldest test for albumen depends upon *its coagulability by heat.* Heat coagulates the serum albumen (opalescence occuring at 60° C., coagulation at 72° to 75°), and also the serum globlin (opalescence occurring at 68° C., coagulation at 75°); has no effect upon the peptones, propeptones, nor upon acid alkali albumen, unless an alkali or acid or has first been added. It, however, produces cloudiness with phosphates, by driving off carbonic acid, which holds them in solution, and the further addition of nitric acid, by redissolving them, clears up the opacity. A preliminary acidulation with acetic or nitric acid prevents this cloudiness, but may convert albumen into acid albumen, and so make the test fail. But on the whole, if cautiously employed, heat will be found a good test. A further security may be obtained by using both acetic acid and a concentrated solution of magnesium sulphate, or of sodic sulphate, or of common salt, for these prevent the undue action of the acid upon the albumen.

The *Cold Nitric Acid Test* ranks next in date of introduction and in general popularity to that by heat. When a layer of nitric acid is brought into contact with a layer of urine, a white coagulum is formed at the line of junction of the fluids. The acid coagulates serum albumen, serum globulin, has no effect upon peptones; gives an opacity with propeptones, which, however, disappears with heat; has no effect upon acid albumen, but gives distinct reaction with alkali albumen. One or two sources of fallacy must be kept in view when one employs this test. It may give a precipitate with urates, with urea, or with resinous substances. Such fallacies may be avoided by the adoption of very simple precautions, which are fully detailed in the books on urinary analysis.

*Metaphosphoric Acid* is an excellent test for albumen, but a sit is only serviceable when pure, and diffcult to keep in that condition, it has not come into general use.

*Acidulated Brine* is also a test of considerable value, acting upon all varieties of albumen, but it is not likely to become greatly trusted, because of its frequently giving some reactions with normal vrine.

*Picric Acid* is a test which has been brought into use in Great Britain mainly by the recommendation of Dr. George Johnson. It produces an opacity with all the forms of albumen; but while those with serum albumen, serum globulin, acid and alkali albumen persist or become more distinct with heat, those with peptone or propeptone dissolve. It must be remembered, also, that alkaloids, such as quinine, give a cloud with this reagent, but one which rapidly disappears with heating. On the whole, I believe this to be the most reliable and delicate test which we at present possess.

It has been objected to the test, that it precipitates mucin as well as serum albumen, and that this is a source of fallacy, particularly when it is used by the contact method. Careful investigations by Prof. Stewart showed that, while a large number of the specimens gave distinct reactions both with picric and citric acids, there were three which gave an opalescence with picric and not with citric, and seven of those which reacted with citric acid gave no reaction with picric. From these facts he concludes that mucin may be demonstrated by citric acid when no reaction is produced with picric, and that picric may show minute quantities of albumen in urines in which citric acid fails to show mucin.

On the other hand, picric acid often produces an opalescence in urine apparently free from albumen, and Dr. Stevens made a series of careful experiments which seem to indicate that picric acid acts upon mucin, although more slowly and less distinctly than does citric acid. The degree of acidity of the urine is probably an important element in relation to this reaction with picric acid; and Prof. Stewart thinks that where acid is present in quantity the opalescence is distinct; where it is in slight amount, it is comparatively or completely absent.

It is likely that although picric acid often affects mucin, it does not do so in such a way as to render it unreliable as a delicate test for albumen. Its precipitate with mucin is, even when applied by the contact method, a slight, slowly developed haze. A precipitate indicating albumen is more marked and more quickly produced. A little practice in the use of the test will soon render any one familiar with the degree and rate of formation of the opacity which indicates albumen as distinguished from those which mark the presence of mucin.

*Potassio-Mercuric-Iodide*, which was first proposed as a test by M. Tanret, corresponds in its action to picric acid, giving opacity with serum albumen, globulin, acid and alkali albumen, and an opacity dissolved by heat with peptone and propeptone. But it will be found to give a reaction with a veiy large proportion of normal urines, and as the addition of an organic acid—citric or acetic—is required to bring out the reaction, it is clear that mucin must, in many cases, give a degree of opalescence. It may be that other sources of fallacy exist in regard to slighter reactions. Dr. Oliver's method of applying this test greatly reduces the chances of error, but its disadvantages render it an inferior test to the picric acid.

*Potassium Ferrocyanide*, first suggested by Dr. Pavy, also resembles picric acid in its action, except it does not give any indication with peptones. The objections which induced Prof. Stewart to reject the reagent last described apply to this one also.

*Dilution with water* is a convenient but not very reliable test of the presence of serum globulin, as it produces a milkiness, that substance being soluble in weak saline solutions, but not in pure water or extremely diluted solutions of salts. It produces no effect upon other forms of albumen.

*Magnesium Sulphate* is a valuable test for serum globulin, as it produces a milky opacity with that substance, which speedily deposits as a precipitate. It has no action upon serum albumen, peptone or propeptone, but produces an opacity with acid and alkali albumen. It is best used in saturated solution by the contact method. By its use also, according to methods described in works dealing with the subject of physiological chemistry, the globulin may be separated nearly pure, and its amount determined.

*Fehling's Solution*, or other alkaline solution of copper, is a most convenient test for peptone and propeptone, giving with these a rose pink or purple color at the point of contact of the test with the supernatant urine, and producing no effect upon the others, with the exception of serum albumen, with which it gives a brownish-red hue.

*Randolph's Test* for peptone and propetone, which consists in the addition of one drop of saturated solution of iodide of potassium and then of two drops of Millon's reagent (an acid solution of nitrate of mercury) to a drachm of urine gives a yellow, instead of a red, precipitate when these substances are present; but, as Randolph has pointed out, it gives the same color reaction with bile salts, which are frequently present in considerable amount in the urine. Therefore we cannot esteem it so highly as the copper and alkali test.

The presence of hæmoglobin may be made out by the guaiac reaction or by the spectroscope; the presence of fibrin may be ascertained by its decomposing with effervescence hydrogen peroxide; mucin may be discovered by means of citric or acetic acid; and waxy material may be shown (if it is ever present) by iodine, and sulphuric acid, or by methylaniline violet.

Table II. I show you the results of tests as to the relative delicacy of the principal tests for albumen. The first column shows the dilution up to which the action of each reagent remained distinct; The second shows the percentage of albumen, as calculated from the total quantity in the undiluted fluid and the number of dilutions; and the third shows the grains, or parts of a grain, per ounce as calculated from the same data:

TABLE II.—*Showing the Comparative Delicacy of Tests for Serum Albumen.*

| Tests. | Dilutions. | Percentage. | Grains per Ounce. |
|---|---|---|---|
| Boiling | 300 | 0.0005 | 0.00218 |
| Acidulation with acetic acid, and boiling | 500 | 0.0003 | 0.001311 |
| Cold nitric acid | 50 | 0.003 | 0.01311 |
| Metaphosphoric acid | 500 | 0.0003 | 0.001311 |
| Picric acid | 1000 | 0.00015 | 0.000655 |
| Potassio mercuric iodide. (Test papers) | 500 | 0.0003 | 0.001311 |
| Ferrocyanide of potassium | 500 | 0.0003 | 0.001311 |

The urine contained albumen to the amount of 1.5 grammes per litre, which is equal to 0.15 per cent., or 0.655 of a grain per ounce.

The results show that the *boiling test*, carefully applied, is an excellent one, revealing the presence of so little as 0.00218 of a grain per ounce, and continuing to show up to the 300th dilution of the standard specimen. But heat, with preliminary acidification with a little acetic acid, was still more delicate, showing 0.001311 of a grain per ounce, and giving a perceptible haziness up to 500 dilutions.

The *Cold Nitric Acid Test* falls far short of this in delicacy, for it does not give a distinct reaction beyond the 50th dilution, and therefore shows only with 0.01311 of a grain per ounce. It is true that if the specimen is allowed to stand, the reaction may gradually manifest itself, with minute traces of albumen; but this is inconvenient, and for practical use tests are to be estimated in proportion to their rapidity of action.

*Metaphosphoric Acid* gave the same results as heating after acidulation with acetic acid, viz., showing till the 500th dilution of the standard urine, and 0.001311 of a grain per ounce.

*Picric Acid* proved the most delicate test, giving a faint but perceptible reaction up to the 1000th dilution of the standard specimen, which is equal to 0.00015 per cent., or 0.000655 of a grain per ounce.

The *Potassio-mercuric Iodide* and the *Ferrocyanide of Potassium Tests* gave the same results as metaphosphoric acid, showing albumen up to the 500th dilution of the standard specimen, equal to 0.0001311 of a grain per ounce.

From these and other observations Prof. Stewart concludes that picric acid is the most delicate of all the reagents which we possess for albumen, and that next to it rank the potassio-mercuric iodide, the heating after acidulation with acetic acid, the ferrocyanide of potassium, and the metaphosphoric acid. Boiling and adding nitric acid is less delicate, and still less so is the cold nitric acid test.

But delicacy is not the only quality required of a test. Indeed, a test may be too delicate for practical purposes. And again tests otherwise suitable may be practically inconvenient. Nitric acid is difficult to carry about, and picric acid presents a similar disadvantage, although in a minor degree. The test pellets devised by Dr. Pavy, of London, and the test papers of Dr. Oliver, of Harrogate, are extremely convenient, being easily carried about, and very delicate. But it may be held that they are too delicate, for few urines fail to show some reaction with them. Indeed, many of them show some reaction with practically normal urines. The smallest quantity of mucin may suffice to produce the reaction, or a quite infinitessimal trace of albumen proper. The tests must be used with discrimination, and too much importance must not be attached to their fainter indications. Albuminuria is rarely a serious condition unless it is sufficiently pronounced to be made out by the cold nitric acid test.

As to the quantitative analysis of albuminous urine, Prof. Stewart approves most highly of the coagulating, drying and weighing process. A less laborious process is that known as Esbach's Method. This plan requires certain special tubes, graduated so as to show the height to which the urine to be tested, and that to which the reagent (a solution of picric and citric acids) should reach, also the number or proportion of grammes per litre. The urine is filled up to the level indicated by the letter U, then the test fluid to the line marked R, and the fluids having been thoroughly mixed, are set aside to stand for twenty-four hours.

At the end of that time the level reached by the coagulum enables us to read oft the grammes per litre. Our observations brought out a result, as is seen in the table, of 2.5 grammes per litre, which is equivalent to 0.25 per cent., or 1.0837 grains per ounce. It thus very closely corresponded to the results odtained by the first method.

Esbach's method brings out results closely corresponding to those obtained by the elaborate drying and weighing process. As the method is easily worked, as well as so reliable, it is a good one to adopt. Its only disadvantages are that one must wait twenty-four hours before the result can be obtained, and that it does not enable us to measure less than 0.5 grammes per litre.—*Quarterly Compendium of Medical Science.*

